# Patients’ Awareness of Their Rights and Responsibilities: A Cross-Sectional Study From Al-Ahsa

**DOI:** 10.7759/cureus.32854

**Published:** 2022-12-23

**Authors:** Meataz K Aljeezan, Yousef Y Altaher, Thamer A Boushal, Anwar M Alsultan, Abdul Sattar Khan

**Affiliations:** 1 Internal Medicine Department, College of Medicine, King Faisal University, Alhasa, SAU; 2 Family and Community Medicine Department, College of Medicine, King Faisal University, Alhasa, SAU

**Keywords:** health-care workers, alhasa, awareness, patients, rights and responsibilities

## Abstract

Introduction

Patient rights are an essential part of healthcare practice. In fact, patients are one of the most vulnerable members of society. As a result, improving the rights of patients is considered a priority in medical services.

Aim

The purpose of this study is to measure the level of patients’ awareness of their rights.

Subjects and methods

This is a cross-sectional study conducted among patients living in Al-Ahsa, Saudi Arabia. A self-administered questionnaire was distributed among Al-Ahsa patients using an online survey. The questionnaire was composed of socio-demographic variables (i.e., age, gender, education, etc.), sources of patient information regarding patients, means of increasing awareness toward patients' rights, and a 15-item questionnaire to measure the awareness about patient rights and responsibilities.

Results

Among the 295 patients, 59.7% were males and 39% were aged between 31 and 40 years old. The overall mean awareness score was 54.6 (SD 7.44). 53.2% of the patients were categorized as having moderate awareness levels, 44.1% were good and only 2.7% were categorized as having poor levels of awareness. Factors associated with increased awareness were being older, hospital admission, hospital visitation for the last three months, and healthcare providers as the sources of the patient's right information.

Conclusion

There was sufficient awareness of patient rights and responsibilities in our region. Increasing age, frequent hospital visitation, and education given by healthcare providers could effectively improve awareness of patient rights and responsibilities. A multicenter study is required to shed more light on the awareness of patients regarding their rights and responsibilities.

## Introduction

In 1948, the United Nations established the Universal Declaration of Human Rights, which has been implemented all over the world [1]. Patient rights are an essential part of healthcare practice. In fact, patients are one of the most vulnerable members of society. As a result, improving the rights of patients is considered a priority in medical services. [2].

Patient rights are an amalgamation of legal and ethical issues concerning the doctor-patient relationship, which includes the patient’s right to privacy, confidentiality, access to quality healthcare services, the right to make informed decisions about treatment options, etc. [3]. Patients’ rights vary in different countries and are influenced by the country-related and social factors of the area [4]. However, in most countries, healthcare organizations have established regulations or charters for patients’ rights and announced and implemented them, in order to achieve patients’ satisfaction [5].

In the Kingdom of Saudi Arabia, the Ministry of Health published the National Patients and their families Rights and Responsibilities booklet, which all patients receive upon hospital admission. The patient bill of rights is a written document that is available in most Saudi healthcare organizations, but many patients and their families may not be aware of their rights that have been granted by the Saudi government through policies and regulations of the Ministry of health [6-8].

Patients’ awareness of their rights can bring about a lot of advantages such as increased quality of health care services, decreased costs, more prompt recovery, decreased length of stay in hospitals, lower risk of irreversible physical and spiritual damages, and more importantly, increased dignity of patients through informing them about their rights to participate in decision making. On the other hand, a lack of respect for patients’ rights may lead to hazards to the security and health situation of patients.

Besides, it may ruin the relationship between the staff and patients which consequently decreases the efficiency, effectiveness, and suitable care of patients [9]. Although some studies have been conducted to determine the level of knowledge about patient rights not only among patients but also among physicians and nurses, there is still a lack of studies that assess the patient's evaluation of the medical services received in light of these rights, particularly in Al-Hasa. As a result, the goals of this study are to assess patients' awareness of their rights, predictors of their knowledge score, and their assessment of whether or not these patients' rights were met when they sought medical advice in the Al-Hasa community.

## Materials and methods

This is a cross-sectional study conducted among patients living in Al-Ahsa, Saudi Arabia. The sample size was 310 calculated by using Cochran's Formula [10] with 95% confidence, a margin of error of 5%, assuming a population proportion of 0.72, and an unlimited population size. All people who live in Al-Hasa and have visited the hospital during the last three months were covered by the study. Individuals under the age of 18 and those who live outside Al-Hasa were excluded. An Arabic-language Google Form was used to distribute the self-administered survey to the target community to boost response rates. The questionnaire was composed of three sections, socio-demographic variables (i.e., age, gender, education, etc.), sources of patient information regarding patients, means of increasing awareness toward patients' rights, and a 15-item questionnaire to measure awareness about patient rights and responsibilities. The PBR section included 15 questions on participants’ awareness of their rights addressing the principal rights of the PBR, scored using a Likert scale (strongly agree, agree, neutral, disagree, or strongly disagree). The questions were scored 1-5 and enquired to what extent patients agreed or disagreed with a specific item of the PBR, with a total score of 15 to 75. Those with higher scores demonstrated better awareness of the PBR. Results were categorized into three levels: poor awareness (15-37), moderate awareness (38-56), and excellent awareness (57-75).

Statistical analysis

The awareness of the patients regarding their rights and responsibilities has been assessed using a 15-item questionnaire with a response of 5-point Likert scale categories ranging from “strongly disagree” coded with 1 and “strongly agree” coded with 5. The total awareness score has been calculated by adding all 15 items. A score ranging from 15 to 75 points had been generated, a higher score indicates a higher awareness of the patient's rights and responsibilities. By using 50% and 75% as the cutoff points to determine the level of awareness, patients were categorized as poor awareness if the score was below 50%, 50% to 75% were categorized as moderate awareness and above 75% were categorized as good awareness levels.

Categorical variables were shown as numbers and percentages (%) while continuous variables were summarized as mean and standard deviation. The differences in the score of awareness in relation to the socio-demographic characteristics of the patients had been performed using Mann-Whitney Z-test and Kruskal Wallis H-test. The normality test was carried out using the Shapiro-Wilk test and Kolmogorov-Smirnov test. The awareness score follows a non-normal distribution. Therefore, the non-parametric test was applied. Two-tailed analyses with p<0.05 were used as the cutoff for statistical significance. All data analyses were performed using the statistical package for social sciences, version 26 (SPSS, IBM Corp., Armonk, NY, USA).

Ethical considerations

Ethical approval was obtained from the Deanship of Scientific Research, Vice Presidency for Graduate Students, and Scientific Research, King Faisal University. Before asking participants to fill out the study questionnaire, the study's aims were explained to them, their informed consent was obtained as well as the study's objectives were described to the participants. They were given the option to accept or decline, and everyone was promised that the data collected would be kept private and anonymous.

## Results

A total of 310 questionnaires were received, 15 of them did not meet the necessary criteria and the remaining 295 questionnaires were eligible subjects giving an overall response rate of 95.2%. Table 1 presented the socio-demographic characteristics of the patients living in Al-Ahsa. The most common age group was 31 to 40 years (39%) with nearly 60% being males. Saudi nationality comprised most of the patients (98%). Patients who had bachelor’s degrees were 59.7%. Approximately 42.7% were earning 5,000 to 15,000 SAR per month. The most common chronic diseases were diabetes (6.8%) and hypertension (6.8%). The most commonly cited reason for hospital admission was pregnancy delivery (7.1%). The proportion of patients who had visited a hospital in the last three months was 77.3%.

**Table 1:** Socio-demographic characteristics of the patients living in Al-Ahsa (n=295)

Study Data	N (%)
Age group	
18 – 30 years	97 (32.9%)
31 – 40 years	115 (39.0%)
41 – 50 years	53 (18.0%)
>50 years	30 (10.2%)
Gender	
Male	176 (59.7%)
Female	119 (40.3%)
Nationality	
Saudi	289 (98.0%)
Non-Saudi	06 (02.0%)
Educational level	
Secondary education	77 (26.1%)
Diploma	26 (08.8%)
Bachelor	176 (59.7%)
Postgraduate	16 (05.4%)
Average monthly income (SAR)	
<5,000	112 (38.0%)
5,000 – 15,000	126 (42.7%)
>15,000	57 (19.3%)
Associated chronic disease	
None	224 (75.9%)
Diabetes	20 (06.8%)
Hypertension	20 (06.8%)
Hypothyroidism	02 (0.70%)
Asthma	09 (03.1%)
Others	20 (06.8%)
Have you ever slept in the hospital for any reason?	
No	188 (63.7%)
Pregnancy Delivery	21 (07.1%)
Surgery	39 (13.2%)
Injury	06 (02.0%)
Comorbidities	16 (05.4%)
Infection and bleeding	10 (03.4%)
Others	15 (05.1%)
Visited hospital/healthcare for the last 3 months	
Yes	228 (77.3%)
No	67 (22.7%)

In Table 2, the most commonly authorized person to view patient medical records without permission was the patient medical team (78.6%). Also, the most commonly known source of patient rights information was hospital administration or patient relations (41.4%) while the most frequently cited means of raising awareness of patients' rights was to make videos or websites and spread them on the internet and on TV (34.6%).

**Table 2:** Authorized personnel to view medical records, sources, and means to increase awareness (n=295) † Variable with multiple response answers.

Statements	N (%)
Who has the right to see your medical record without your permission? ^†^	
My work manager	27 (09.2%)
My medical team	232 (78.6%)
The hospital quality management program team	55 (18.6%)
People with written authorization from the patient or legal authorities	156 (52.9%)
The hospital health facility research team	74 (25.1%)
What is the primary source of patient rights information?	
Old Media (TV - newspaper- radio)	10 (03.4%)
Doctor, nurse, or other health care provider	67 (22.7%)
New media e.g., social media on the Internet	66 (22.4%)
Booklet, brochure, or poster boards	16 (05.4%)
Family or friends	14 (04.7%)
Hospital administration or patients relations	122 (41.4%)
Which of the following statements is most effective in raising awareness of patients' rights among the population, in your opinion?	
Increase leaflets and booklet in the waiting area	27 (09.2%)
Make videos or websites and spread them on the internet and on TV	102 (34.6%)
Special Office in every hospital for patients’ rights education	69 (23.4%)
Include this topic in school and university curriculum	47 (15.9%)
Educate and evaluate the healthcare providers to respect patients’ rights	50 (16.9%)

 

In Table 3, the top three statements where the patients expressed the highest ratings were “Healthcare centers should inform patients of their rights in a way that they can understand” (mean score: 4.67), “The physician must respect the patient's cultural, spiritual, and religious values” (mean score: 4.63), and “The patient has the right to know the identity and professional status of health care providers (nurses and doctors)” (mean score: 4.43). The mean total awareness score was 54.6 (SD 7.44) with poor, moderate, and good awareness levels found among 2.7%, 53.2%, and 44.1%, respectively.

**Table 3:** Assessment of patient's awareness regarding their rights and responsibilities (n=295) Response has ranged from “strongly disagree” coded with 1 to “strongly agree” coded with 5. Reverse-coded question.

Awareness statement	Mean ± SD
Healthcare centers should inform patients of their rights in a way that they can understand	4.67 ± 0.86
The physician must respect the patient's cultural, spiritual, and religious values	4.63 ± 0.89
The patient has the right to know the identity and professional status of health care providers (nurses and doctors)	4.43 ± 1.01
A patient was newly diagnosed with a sexually transmitted infection, and the doctor told the patient's wife to prevent transmission of the infection to her. Do you agree with the doctor's action?	4.34 ± 1.19
The patient is not entitled to register a complaint or oral or written suggestion to the health center ^†^	4.27 ± 1.31
A doctor must evaluate and treat a patient's pain, even if he or she has several patients in the clinic	4.21 ± 1.13
The patient is responsible for his or her decision if he or she refuses medical care advice	4.12 ± 1.30
The doctor does not have to tell the patient everything about his or her health, such as complications or negative effects of medication ^†^	4.07 ± 1.40
The patient is not entitled to a second opinion ^† ^	4.03 ± 1.30
The hospital has the right to stop caring for the patient if he refuses treatment	3.02 ± 1.55
The medical team has the right to provide appropriate medical or surgical interventions without the consent of the patient	2.66 ± 1.47
The doctor is the only person who can determine the correct examination and treatment plan, and the patient does not have the right to express an opinion	2.65 ± 1.53
The patient should participate in any research studies conducted in the hospital	2.63 ± 1.49
A diabetic patient refuses to take his medication, so the doctor gave him diabetes medication and convinced him that it was vitamins. Do you agree with the doctor's actions?	2.44 ± 1.57
In the emergency room, Saudi patients are treated before patients of other nationalities	2.38 ± 1.61
Total Awareness score (mean ± SD)	54.6 ± 7.44
Level of awareness	
Poor	08 (02.7%)
Moderate	157 (53.2%)
Good	130 (44.1%)

When measuring the differences in the score of awareness according to the socio-demographic characteristics, sources, and means of increasing awareness of patient's rights and responsibilities (Table 4), it was observed that a higher awareness score was more associated with being older in age (H=6.076; p=0.048), hospital admission (Z=2.871; p=0.004), hospital visitation for the last three months (Z=2.173; p=0.030), healthcare providers as the sources of patient right information (H=13.596; p=0.018) and perceived effective means of increasing awareness by making videos or websites (H=10.676; p=0.030). No significant differences were observed regarding the awareness score in terms of gender, educational level, average monthly income, and associated chronic disease (p>0.05).

**Table 4:** Differences in the score of awareness in relation to the Socio-demographic characteristics, sources, and means of increasing awareness of patient's rights and responsibilities (n=295) a P-value has been calculated using Kruskal Wallis H-test. b P-value has been calculated using Mann-Whitney Z-test. ** Significant at p<0.05 level.

Factor	Awareness Score (75) Mean ± SD	Z/H-test	P-value
Age group			
18 – 30 years	53.5 ± 7.45	H=6.076	0.048 ** ^a^
31 – 40 years	54.2 ± 8.02		
>40 years	56.3 ± 6.27		
Gender			
Male	54.4 ± 7.67	Z=0.551	0.581 ^b^
Female	54.9 ± 7.11		
Educational level			
Diploma or below	54.4 ± 8.27	Z=0.280	0.779 ^b^
Bachelor or higher	54.7 ± 6.97		
Average monthly income (SAR)			
<5,000	54.9 ± 7.40	H=1.995	0.369 ^a^
5,000 – 15,000	54.9 ± 7.41		
>15,000	53.3 ± 7.58		
Associated chronic disease			
Yes	54.3 ± 6.79	Z=0.633	0.526 ^b^
No	54.7 ± 7.64		
Admitted to hospital			
Yes	56.4 ± 5.64	Z=2.871	0.004 ** ^b^
No	53.5 ± 8.12		
Visited hospital/healthcare for the last 3 months			
Yes	55.1 ± 7.20	Z=2.173	0.030 ** ^b^
No	52.9 ± 8.04		
Sources of patients' right information			
Old Media (TV - newspaper- radio)	53.4 ± 6.70	H=13.596	0.018 ** ^a^
Doctor, nurse, or other health care provider	55.9 ± 8.74		
New media e.g., social media on the Internet	55.7 ± 7.97		
Booklet, brochure, or poster boards	53.4 ± 6.62		
Family or friends	51.4 ± 9.39		
Hospital administration or patients relations	53.8 ± 6.06		
Most effective means of raising awareness of patients' rights			
Increase leaflets and booklet in the waiting area	53.8 ± 9.38	H=10.676	0.030 ** ^a^
Make videos or websites and spread them on the internet and on TV	55.9 ± 6.84		
Special Office in every hospital for patients’ rights education	53.1 ± 6.74		
Include this topic in school and university curriculum	54.4 ± 6.83		
Educate and evaluate the healthcare providers to respect patients’ rights	54.6 ± 8.67		

## Discussion

This study examined the awareness of the patients regarding their rights and responsibilities during hospital visitation or admission. Based on the given criteria, the overall awareness level of our patients was adequate. Approximately, 53.2% were estimated to have moderate awareness levels, 44.1% were good and only 2.7% were estimated to have poor levels (mean score: 54.6; SD 7.44, out of 75 points). These findings are consistent with the study of Unnikrishnan et al. [3]. According to their reports, nearly 60% of the patients demonstrated moderate awareness, 10.1% were good and 30% demonstrated weak levels. Similarly, in Riyadh [11], most patients were moderately aware of their rights (72.2%), however, 65.3% exhibited a lack of knowledge about the existence of patients' bill of rights. Contradicting these reports, several studies documented a lack of awareness of patients regarding their rights [6,12-15]. Appropriate measures have to be taken to maintain the quality of health care practice, and control and eradicate the factors that lead to abuse of patient's rights.

The results of this study indicate that increasing age was associated with an increasing level of awareness. This contradicted the report of Agrawal et al. [16]. According to their survey, younger adults were more aware than older ones while female patients had a higher level of awareness than their male counterparts. The influence of gender on the awareness of patient rights had also been documented by Unnikrishnan et al. [3]. Accordingly, they found that the awareness of patient rights was independently associated with gender, socioeconomic status, and educational levels with similar findings as reported by Al-Rebdi et al. [11], as well as Almalki et al. [17]. In our study, however, the awareness levels in terms of gender, education, and socioeconomic status were comparable across the groups which did not coincide with previous reports.

Likewise, better awareness levels were predicted to be seen more frequently by the patients who had regular visits to the hospital. In Pakistan [13], citing dissatisfaction with services among public hospitals, patients who sought care in private hospitals demonstrated a better awareness level in terms of rights and responsibilities while among Indian patients [16], those who were admitted in wards or higher levels (deluxe room) had a better degree of awareness about patient rights and education. The author then noted that it is important for the stakeholders to devise a plan to improve awareness not only among patients but also among healthcare providers to deliver the optimum quality of care to its client.

Discussing the specific determinants of awareness, according to participants' ratings (out of 5 points), most of the patients were highly aware that it is the healthcare center's responsibility to educate patients about their rights and that the information should be easy to understand (mean score: 4.67), they emphasized that physician must respect their cultural, spiritual and religious values (mean score: 4.63), and they were aware of their right to know the identity and professional level of the nurses or doctors (mean score: 4.43). On the contrary, patients were less aware that the doctor can give false information about the medication just to convince the patient to take the drugs (mean score: 2.44) and they were unaware that some Saudi patients may have been prioritized in the emergency room than other nationalities (mean score: 2.38). A similar report had also been documented in Malaysia [18]. Accordingly, they found out that nearly all patients (99%) indicated that their religious beliefs were respected by the staff and they had no problems in doing their regular religious routine. Furthermore, informed consent was obtained regularly, in most cases by the doctor (98%) and the rest by the nurses (2%) adding that 98% of the patients expressed that their treatments/examinations were conducted in a private room with personal identity was kept confidential by their doctors. In Iran [5], patients had given the highest rating toward the trust and assurance of confidentiality of the treatment team while the least was about providing sufficient information about the treatment options and their complications.

The importance of sources of information regarding patients' rights and the ways to increase awareness were also tackled in this study. In this regard, the most commonly known sources of patient rights information were hospital administration or patient relations (41.4%), healthcare providers such as doctors and nurses (22.7%), and social media (22.4%) while mass media (TV, newspaper, and radio) was the least source of the patients. We also observed that having healthcare providers as sources of patient rights information significantly improved awareness levels. Healthcare providers as the main sources of patient rights information had been well documented in the literature with social media being the secondary source [3,6,11]. Surprisingly, in South Africa [14], patients demonstrated a lack of awareness about the Patients’ Rights Charter, and they were unable to give examples or indicate the rights they have as patients. Lack of sources of information was cited as the main challenge by the patients in getting adequate education about their rights.

Moreover, in terms of the effective means of raising awareness, our patients indicated that the most viable ways were about making videos or websites and posting them on the internet or TV (34.6%). Data in our study further suggest that this method could lead to a significant improvement in citizens’ awareness of their rights. This was followed by a stationed office in every hospital to address the lack of education about patients’ rights (23.4%) and providing education and evaluation of the healthcare providers in relation to giving respect to patients' rights (16.9%), other mentioned means were inclusion of this topic at school or university curriculum (15.9%) and increase promotion through leaflets and booklet in the waiting area (9.2%). Although our patients were aware of the means to increase awareness about patients' rights, on the other hand, among Sudanese patients, the most commonly practiced rights at their hospitals were; the right to be asked for permission before undergoing the screening (88.1%), proper handling (87.8%), the safety of the hospital (87%), presence of a third person when screening a female by a male doctor (85.6%), and admission file confidentiality (75.5%) while among Saudi patients [17], the most common challenges reported by patients leading to inadequate awareness of their rights and responsibilities were poor patients' education programs (63.2%), poor physician-patient cooperation (37.4%), lack of hospital staff (30.2%) and staff’s overwhelming workload and lack of time (23.4%).

## Conclusions

There was sufficient awareness of patient rights and responsibilities in our region. Increasing age, frequent hospital visitation, and education given by healthcare providers could effectively improve awareness of patient rights and responsibilities. Although most of our patients demonstrated adequate awareness, however, there is still room for improvement and attempts should be exerted to improve it. Continuous efforts should be made to properly inform patients regarding their rights, and what to expect from the services provided by the hospitals and healthcare providers. The patient rights charter should be readable and easy to understand. In addition, the development and dissemination of educational materials in simple language about the rights and responsibilities of the patient and their family/relatives during their stay in the hospital or at the time of registration are vital to make them fully aware of their rights. Ultimately, a committee to oversee patient's rights are followed must be formed to ensure observance of the rights of the patient.
